# Reduction of impulsivity in patients receiving deep transcranial magnetic stimulation treatment for obesity

**DOI:** 10.1007/s12020-021-02802-1

**Published:** 2021-06-25

**Authors:** Livio Luzi, Sara Gandini, Stefano Massarini, Federica Bellerba, Ileana Terruzzi, Pamela Senesi, Concetta Macrì, Anna Ferrulli

**Affiliations:** 1 Department of Biomedical Sciences for Health, University of Milan, Milan, Italy; 2 Department of Experimental Oncology, European Institute of Oncology IRCCS, Milan, Italy; 3 Present Address: Department of Endocrinology, Nutrition and Metabolic Diseases, IRCCS MultiMedica, Sesto San Giovanni, Milan, Italy

**Keywords:** Obesity, Transcranial Magnetic Stimulation, Psychological traits, Impulsivity, Leptin

## Abstract

**Purpose:**

Aims of the present study were to investigate a wide array of psychological symptoms through validated psychometric tests, before and after 5 weeks of deep Transcranial Magnetic Stimulation (dTMS) in individuals with obesity, and to identify possible relationships with neuroendocrine parameters.

**Methods:**

Forty-five patients with obesity (33 F, 12 M; age 48.8 ± 9.9 years; body wt 97.6 ± 14.2 Kg; BMI 36.2 ± 4.2) were randomized into two groups: 26 received high frequency (HF) dTMS and 19 Sham stimulation for 5 weeks. At baseline and after the 5-week treatment, all patients underwent the following psychometric evaluations: Food Cravings Questionnaire-Trait (FCQ-T) and its subscales, Barratt Impulsiveness Scale-11 (BIS-11), State and Trait Anxiety Inventory (STAI-y1 and STAI-y2), and Beck Depression Inventory (BDI). Hormonal and neuroendocrine markers were assessed at the first and last dTMS session.

**Results:**

By adjusting for baseline variables and treatment arms, a significant decrease in body wt and BMI was found in HF group, both with univariate (*p* = 0.019) and multivariate analyses (*p* = 0.012). Impulsivity significantly decreased in HF group, both with univariate (*p* = 0.031) and multivariate analyses (*p* = 0.011). A positive association between the impulsivity score change and the leptin level variation (*p* = 0.031) was found.

**Conclusion:**

The decrease of impulsivity together with the BMI reduction in individuals with obesity, treated with real stimulation, suggests that impulsivity may be a risk factor for obesity. Treatment with dTMS revealed to be effective in reducing both BMI and impulsivity by enhancing inhibitory capacity of Pre-Frontal Cortex (PFC), and modulating neuroendocrine system, especially leptin.

## Introduction

In the past 50 years the prevalence of obesity worldwide has nearly tripled, reaching pandemic proportions and becoming a global health concern [1]. A long-term energy imbalance between too many calories consumed and too few calories expended has been commonly identified as the fundamental cause of obesity. Notwithstanding, the main strategies for the treatment of obesity, aimed at reducing energy intake and increasing exercise, are frequently not successful, suggesting a more complex aetiology underlying obesity [1]. In fact, numerous other factors could affect the chronic positive energy balance in obesity: age, sex, genetics, neuroendocrine factors, gut microbiota, concomitant medications, socio-cultural level, lack of knowledge, homeostatic hunger, uncontrolled eating, and emotional eating [1].

Several studies have contributed at identifying a bidirectional relationship between obesity and psychological symptoms, not necessarily entailing a psychiatric diagnosis according to Diagnostic and Statistical Manual of mental disorders 5 (DSM-5) criteria. Psychological traits may be at the same time both a risk factor for obesity, and a consequence of the latter.

Anxiety, the most common psychiatric disorder in the developed world [2], has been hypothesized to be a risk factor for obesity. An exhaustive metanalysis highlighted a moderate level of evidence for a positive association between anxiety disorders and obesity, which is stronger in severe obesity (BMI ≥ 35) and in the female sex [3]. In most cases people with obesity develop a form of social anxiety, due to the concern that they will embarrass themselves and that people won’t accept or value them [4]. At the same time, individuals with obesity with more social anxiety symptoms exhibit higher inflammation levels and greater insulin resistance [5], suggesting an increased susceptibility of the more anxious individuals to develop obesity.

Healthcare providers who treat obesity are undoubtedly aware also of the high prevalence of mood disturbances among their patients. It has been estimated that people with obesity have a 55% increased risk of developing depression over time, whereas individuals with depression have a 58% increased risk of becoming obese [6].

Another phenotype trait that may play a critical role in the aetiology of obesity is the impulsivity, defined as “a predisposition toward rapid, unplanned reactions to internal or external stimuli without regard to the negative consequences of these reactions” [7]. Several studies on impulsivity have shown that obesity is associated with less effective inhibitory control [8], assuming a possible dysfunction of the prefrontal cortex (PFC), which is generally implicated in high-order executive function, regulation of limbic reward regions, and inhibition of impulsive behaviors [9]. In fact, in individuals with obesity, a decreased activation of PFC leading to an impaired executive functioning and poorly regulated appetite control behaviors has been observed [9].

While several studies indicate that some psychological traits are associated with disordered eating [10] and weight gain [11], the mechanisms underlying the relationships between these factors have not yet been exhaustively investigated. Hunger and satiety hormones such as ghrelin and leptin, and their involvement in the suppression activity of stress responses by Hypothalamic-Pituitary-Adrenal (HPA) axis are hypothesized to play a role in the relationship between appetite/weight changes and psychological disorder onset [12]. Elevated levels of leptin have been associated with obesity, psychological distress, increasing systemic inflammation, increasing risk in the development of several maligniances (colon, ovarian cancer, prostate, and breast cancers) [13]. Furthermore, the chronic hyperactivation of the HPA axis observed in obesity could derive from the individual inability to cope with long-term enviromental stressful events, by impacting at the same time on autonomic and neuroendocrine outflow, and on behavior [14].

The bidirectional relationship between obesity and anxiety/mood disorders is also based on the dysregulation of brain networks implicated in emotion regulation, reward processing and cognitive control [6], as well as in the homeostatic regulation of food intake [15].

In a recent clinical trial, we demonstrated the efficacy of deep Transcranial Magnetic Stimulation (dTMS), a non-invasive neurostimulation technique, based on the principle of electromagnetic induction, in controlling food craving and reducing body wt up to 1 year period in individuals with obesity [16]. The deep TMS is characterized by the use of coils (double-cone coil, Halo coil, and H-coil), which allow stimulating brain regions up to 4.5–5.5 cm from the skull (vs 1.5 cm of the standard coils). Using traditional TMS with circular or figure of eight coils, regions of deep brain cannot be reached, and much higher stimulation amplitudes are needed to stimulate them [17]. As a possible mechanism of the dTMS, we recently demonstrated that excitatory stimulation of the bilateral PFC and of the deeper insula, via high frequency (HF) dTMS, increases the whole-brain functional connections of the medial orbitofrontal cortex belonging to the PFC, and decreases the whole-brain functional connections with the occipital pole, diminishing reactivity to bottom-up visual-sensory processes in favor of increased reliance on top-down decision-making processes. These effects result in an enhanced PFC inhibitory capacity and thereby, an improved control on eating behavior [18].

In addition to its effects on the PFC, dTMS also affects structures to which the PFC projects, modulating several neurotransmitter systems (serotonin, dopamine, GABA, glutamate, endorphins) [19, 20]. Modulation of these systems represents the main mechanism through which TMS revealed to be an effective treatment for depression.

Based on the above-mentioned evidences, we hypothesized that active stimulation of bilateral PFC and insula by repetitive dTMS in patients with obesity could influence psychological traits, other than food craving and body wt control. The aim of this study was to realize an exhaustive analysis of psychological traits associated with obesity (anxiety, depression, impulsivity, different aspects of food craving) before and after a 5-week treatment with HF dTMS, and compare the effects of dTMS with a control group receiving the Sham stimulation. Furthermore, possible relationships between psychological traits and neuroendocrine parameters modifications have been investigated.

## Methods

### Study setting

This study was performed at the Endocrinology and Metabolic Diseases Division, IRCCS Policlinico San Donato, San Donato Milanese (MI), Italy.

Original study protocol was designed as a double-blind, sham-controlled, randomized clinical trial aimed at investigating the effects of a 5-weeks treatment with dTMS in reducing food craving and body wt in subjects with obesity, comparing HF (HF, 18 Hz) with low frequency (LF, 1 Hz) stimulation and with Sham. The trial has been registered with ClinicalTrials.gov, number NCT03009695.

In 2019, we published preliminary results of the study, demonstrating the safety and efficacy of dTMS, in addition to physical exercise and hypocaloric diet, in reducing body wt for up to 1 year in people with obesity (16). In this study, statistical analysis highlighted poor efficacy of low-frequency stimulation in controlling food craving and reducing body wt in obesity. Therefore, after approval of a protocol amendment by the Ethics Committee, we discontinued recruitment to the LF group, and only enrolled in the HF and Sham groups.

### Study approval

The study was conducted in accordance with the 1964 Helsinki declaration, and it received approval from the local institutional review boards (Ethics Committee of San Raffaele Hospital, Milan, Italy) in the amended version (Version Nr.3) dated 06/10/2016 (protocol number: 27778, file # 137498928 #). All participants provided written informed consent before participating in any study procedures.

### Study participants

Adult men and women (aged 22–65 years, inclusive), who referred to the Endocrinology and Metabolic Diseases outpatient clinic for overweight/obesity treatment from January 2017 to January 2020, were screened with a short interview to determine eligibility. Patient recruitment strategy involved direct interviews. Inclusion and exclusion criteria are reported in Table 1.

**Table 1:** Inclusion and exclusion criteria of participants

Inclusion criteria	Exclusion criteria
Age 22–65 years	Personal or a family history of seizures
BMI 30–45 Kg/m^2^	Organic brain disorders
Willingness to reduce body weight	Psychiatric disorders according to DSM-5 criteria
	Implanted metal devices
	Fasting blood glucose level >150 mg/dl
	Abuse of substances other than nicotine
	Weight variation (>3%) within 3 months prior the screening visit
	Current or recent (within 6 months prior the screening visit) treatment with anti-obesity medications or other medications for body weight reduction
	Medications associated with lowered seizure threshold
	Type 1 diabetes or insulin-treated type 2 diabetes

*BMI* Body Mass Index, *DSM* Diagnostic and Statistical Manual of Mental Disorders

### Randomization and masking

**Fig. 1:**
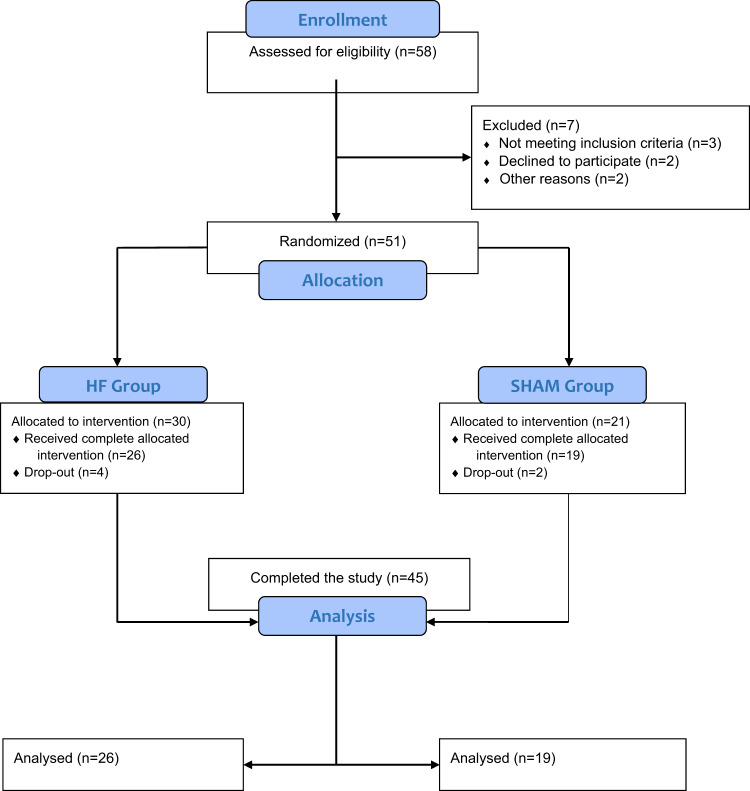
Participant flow chart. CONSORT diagram showing the flow of patients through each stage of the randomized, controlled trial

Patients fulfilling all inclusion/exclusion criteria were randomized to one of two experimental groups: HF or Sham. Allocation in the two groups was performed according to a randomization sequence generated by a computerized program. The study design is shown in the flow chart (Fig. 1). The randomization code was only given to the treating investigator at the first treatment session by an independent investigator not involved with any other aspect of the trial. Participants and other investigators were unaware of the type of treatment assignment. Magnetic cards encoding for real or sham stimulation were used to activate the dTMS device or not, according to the randomization sequence.

### Intervention

Repetitive dTMS was applied using a Magstim Rapid^2^TMS (The Magstim Co. Ltd., Whitland, Carmarthenshire, United Kingdom) stimulator equipped with an H-shaped coil, specifically targeted to bilaterally stimulate the PFC and the insula [21]. For active stimulation, sessions consisted of 80 trains of 18 Hz, each lasting 2 s, with an intertrain interval of 20 s. The HF treatment duration was 29.3 min with 2880 pulses in total. Sham stimulation entailed the same coil placement and procedures as the active condition; however, the device automatically turned off after 15 s of active stimulation, producing similar acoustic artefacts and scalp sensations. Each patient received a total of 15 treatment sessions, 3 times per week in 5 weeks (visit 1–15).

### Psychometric assessments

During the screening period (T0) and after 5 weeks of dTMS or Sham stimulation (T1), all patients enrolled in the study underwent the following psychometric evaluations:

- Food Cravings Questionnaire-Trait (FCQ-T) and its subscales: this test was used to assess both a total measure of trait craving and nine dimensions of food craving [22]. The nine food craving’s dimensions assessed by the FCQ-T are specified in the Supplementary Information;
- Barratt Impulsiveness Scale-11 (BIS-11): this scale has been used to assess impulsivity [23];
- State and Trait Anxiety Inventory (STAI): this test was used to assess state (STAI-*y*1) and trait (STAI-*y*2) anxiety [24];
- Beck Depression Inventory (BDI): this test has been employed to measure depression [25].

A detailed description of the psychometric tests features is shown in the Supplementary Information.

### Laboratory measurements

The following metabolic, hormonal and neuroendocrine markers: glucose (mg/dL), insulin (μU/mL), glucagon (pg/mL), leptin (ng/mL), total ghrelin (ng/mL), β-endorphins (ng/mL), epinephrine (pg/mL), norepinephrine (ng/mL), prolactin (ng/mL), Thyroid-Stimulating Hormone (TSH) (µUI/mL), salivary cortisol (µg/dL) were assessed at T0 and T1.

Details of laboratory measurement procedures have been reported in the Supplementary Information.

#### Statistical analysis

Descriptive statistics were provided for all variables. Continuous variables were presented as median and interquartile ranges. Nonparametric Wilcoxon-rank tests and multivariable adjusted regression models for difference among arms of biomarkers changes adjusted for baseline values were applied to investigate effect of intervention on biomarkers changes in time. Multivariable Generalized linear regression models were also applied to investigate the associations of biomarkers with BIS changes, adjusting for baseline value and treatments arms. Normal distributions of fully adjusted models were graphically checked.

## Results

Out of the 51 initially randomized patients (30 in HF, 21 in Sham), six patients dropped out from the study and were excluded from the statistical analysis. Details about the the dropped-out patients are shown in the Supplementary Information.

A total of forty-five patients with obesity (33 F, 12 M; age 48.8 ± 9.9 years; body wt 97.6 ± 14.2 Kg; BMI 36.2 ± 4.2) completed the study as per protocol and underwent the psychometric assessment at baseline and after 5 weeks of HF dTMS or Sham stimulation. Out of 45 patients, 26 were enrolled in HF and 19 in Sham. At baseline, no significant differences were observed for socio-demographic characteristics and examined parameters, between the two groups. Males were 30.7% in HF and 21.1% in Sham (*p* = 0.47). Median age was in Sham 52 (IQR: 42; 58) and in HF 48 (IQR: 42; 55), and the difference was not statistically significant (*p* = 0.41). Body wt and BMI medians in both groups are reported in Table 2.

**Table 2:** Median and interquartile range of biomakers by intervention arm and time

Intervention arm	*n*.	Variable	Median T0	Lower quartile	Upper quartile	Median T1	Lower quartile	Upper quartile	Change median	Lower quartile	Upper quartile	*P* value^a^	*P* value^b^
HF	26	Body weight	94	86.7	103.9	90.65	81.9	101.6	−3.2	−4.4	−2.2	0.019	0.012*
		BMI	34.3	32.7	37.3	33.5	31.2	36.2	−1.125	−1.7	−0.8	0.020	0.013*
		Glucose	86	77	101	84	82	101	−1	−5	6	0.934	0.764
		β-Endorphins	0.452	0.259	0.58	0.454	0.199	0.544	0.001	−0.11	0.08	0.725	0.915
		Epinephrine	508.641	128.11	814.24	346.307	124.953	866.67	14.28	−80.9	74.318	0.104	0.831
		Norepinephrine	4.4659	3.19	5.5	3.5838	2.8	4.2	−0.345	−1.9	0.746	0.156	0.150
		Insulin	13.865	9.82	23.11	11.305	8.26	16.76	−1.145	−4.02	1.49	0.453	0.858
		Glucagon	40.7	33.6	45.3	40.8	35.7	46.6	−0.15	−4.2	3.85	0.562	0.404
		Total ghrelin	5.836	2.74	10.705	5.162	2.66	12.317	0.05	−1.58	1.94	0.615	0.553
		Leptin	62.2175	37.29	87.52	45.7445	18	63	−12.65	−30.7	0.465	0.489	0.694
		TSH	2.72	1.76	3.39	2.4	1.37	3.23	−0.22	−0.82	0.37	0.794	0.477
		Prolactin	16.535	11.93	20.81	14.24	12.47	19.46	−1.075	−2.95	1.72	0.991	0.203
		Cortisol	0.4	0.28	0.52	0.4	0.23	0.58	0	−0.1	0.083	0.417	0.061
Sham	19	Body weight	96.7	89	106.7	94.7	87.2	105	−1.8	−2.9	−0.7		
		BMI	36.2	34.9	38.7	36.4	33.69	38	−0.7	−1.1	−0.3		
		Glucose	88	82	106	93	87	100	−0.5	−9	7		
		β-Endorphins	0.507	0.432	0.605	0.495	0.393	0.63	−0.021	−0.079	0.01		
		Epinephrine	355.69	139.93	646.2	489.74	310.69	892.79	52.71	−11.27	163.05		
		Norepinephrine	3	1.64	4.51	3.71	1.919	4.735	0.05	−0.345	0.965		
		Insulin	13.32	11.52	22.12	14.705	8.64	20.2	−3.02	−6.13	0.12		
		Glucagon	36.9	28.4	45.7	40.4	31.4	49.2	2.2	−4.4	6.8		
		Total ghrelin	4.04	2.68	9.66	7.9	6.4	12.21	0.34	−1.7	5.99		
		Leptin	59.27	30.66	77.95	37.84	29.15	66.88	−13.15	−23.78	7.22		
		TSH	2.75	1.99	4.69	2.99	1.9	3.64	−0.05	−1.36	0.51		
		Prolactin	15.15	11.44	16.74	13.055	11.53	16.29	−0.255	−2.77	2.32		
		Cortisol	0.37	0.23	0.58	0.33	0.22	0.41	−0.06	−0.16	0.05		

*T0*: At the screening period; *T1*: after 5 weeks of dTMS or Sham stimulation

*HF* High Frequency group, *BMI* Body Mass Index, *TSH* Thyroid-Stimulating Hormone

^a^*P* values from wilcoxon-rank tests

^b^*P* values from multivariable adjusted regression models for difference among arms of biomarkers changes adjusted for baseline values.

*p < 0.05

### Body wt and BMI

By adjusting for baseline variables and treatment arms, a significant decrease in body wt was found in HF group [median 94 (IQR: 86.7; 103.9) vs median 90.65 (IQR: 81.9; 101.6); median change −3.2 (IQR: −4.4; −2.2)], both with univariate (*p* = 0.019) and multivariate analyses (*p* = 0.012).

**Fig. 2:**
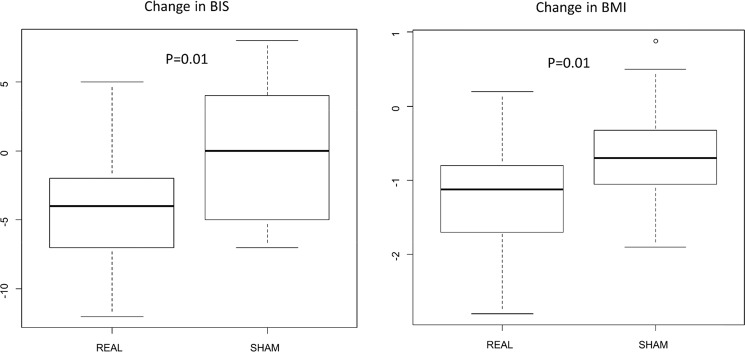
Box plot of changes in time of BIS and BMI by trial arms. *P* value from Wilcocon rank test. *BMI*: Body Mass Index. *BIS-11*: Barratt Impulsiveness Scale-11

Consistently with body wt, a significant decrease in BMI was found in HF group [median 34.3 (IQR: 32.7–37.3) vs median 33.5 (IQR: 31.2; 36.2); median change −1.125 (IQR: −1.7; −0.8)], both with univariate (*p* = 0.02) and multivariate analyses (*p* = 0.012) (Table 2 and Fig. 2).

### Neuroendocrine parameters

Univariate and multivariate analyses did not show significant variations in glucose, insulin, glucagon, leptin, total ghrelin, β-endorphins, epinephrine, norepinephrine, prolactin, TSH, salivary cortisol levels after the 5-week treatment in both the groups (Table 2).

### Psychometric assessments

A significant decrease in BIS-11 score was found in HF group [median 60.5 (IQR: 54; 69) vs median 57 (IQR: 50; 63); median change −4 (IQR: −7; −2)], both with univariate (*p* = 0.031) and multivariate analyses (*p* = 0.011) (Table 3 and Fig. 2).

**Table 3:** Median and interquartile range of psychometric tests by intervention arm and time

Intervention arm	*n*.	Variable	Median T0	Lower quartile	Upper quartile	Median T1	Lower quartile	Upper quartile	Change median	Lower quartile	Upper quartile	*P* value^a^	*P* value^b^
HF	26	STAI-*y*1	43	35	58	36	30	49	−3	−9	0	0.671	0.747
		STAI-*y*2	42	35	52	38.5	27	49	−3.5	−8	−1	0.418	0.297
		BDI	4.5	4	10	3	2	8	−1	−3	0	0.551	0.697
		BIS-11	60.5	54	69	57	50	63	−4	−7	−2	0.031	0.012*
		FCQ-T	121	92	144	75.5	55	108	−47	−58	−12	0.927	0.631
		ANT_pos	16	13	20	11	7	15	−5	−9	−1	0.927	0.518
		ANT_neg	9.5	6	11	6.5	4	9	−2	−3	0	0.854	0.577
		Intent	9	8	11	5.5	3	7	−3.5	−5	−1	0.908	0.530
		Cues	14	11	17	8.5	7	12	−5	−6	−3	0.837	0.451
		Thoughts	18.5	9	23	9	8	14	−5.5	−11	−1	0.349	0.943
		Hunger	12.5	11	16	8.5	6	12	−3.5	−7	0	0.909	0.126
		Control	21.5	14	24	11	7	16	−8	−12	−3	0.882	0.685
		Emotions	12	8	18	8	5	12	−4	−6	−1	0.927	0.888
		Guilt	9	6	13	7	4	9	−3	−5	0	0.943	0.670
Sham	19	STAI-*y*1	40	34	48	34.5	33	40	−3.5	−8.5	1		
		STAI-*y*2	38.5	35.5	47.5	36	31	44	−3	−6	0.5		
		BDI	4	4	7	4	2	6	0	−3	2		
		BIS-11	60.5	54	65	57	56	64	0	−5	4		
		FCQ-T	102	86	153	72	57	87	−28	−68	−14		
		ANT_pos	16	11	22	10	8	13	−4	−10	−1		
		ANT_neg	9	6	14	5	4	9	−1	−6	0		
		Intent	8	6	11	5	4	6	−3	−6	−1		
		Cues	15	10	17	8	6	10	−4	−10	−1		
		Thoughts	13	9	18	8	7	13	−2	−9	0		
		Hunger	11	9	16	7	5	10	−3	−8	−1		
		Control	18	13	26	11	7	13	−6	−15	−3		
		Emotions	13	9	17	8	6	12	−4	−9	−1		
		Guilt	10	7	14	8	5	10	−3	−5	1		

*T0*: At the screening period; *T1*: after 5 weeks of dTMS or Sham stimulationre was found in HF group [median 60.5 (IQR

*HF* High Frequency group, *BMI* Body Mass Index, *STAI* State and Trait Anxiety Inventory, *BDI* Beck Depression Inventory, *BIS-11* Barratt Impulsiveness Scale-11, *FCQ-T* Food Cravings Questionnaire-Trait, (*Ant* +) anticipation of positive reinforcement as a result from eating, (*Ant*−) anticipation of relief from negative states and feelings as a result from eating

^a^*P* values from wilcoxon-rank tests

^b^*P* values from multivariable adjusted regression models for difference among arms of biomarkers changes adjusted for baseline values

*p < 0.05

### Relationships between BIS-11 variation and neuroendocrine parameters changes

Multivariate generalized linear regression model showed a significant direct association between the BIS-11 score change and the leptin level variation: at increasing values of leptin, significant greater change in BIS-11 corresponds (est 0.057, StErr 0.025, *p* = 0.031), adjusting for baseline values and treatments arms (Table 4).

**Table 4:** Multivariable generalized linear regression model investigating the associations of biomarkers with BIS changes, adjusting for baseline value and treatments arms

	Biomarker at baseline			Biomarker change		
	est	StErr	*P* value	est	StErr	*P* value
Intercept	−3.711	7.972	0.644	5.693	4.939	0.256
BIS-11 at baseline	−0.024	0.081	0.770	−0.088	0.078	0.266
Body weight	0.049	0.050	0.337	0.511	0.317	0.114
HF vs Sham	−3.665	1.364	0.010	−2.627	1.465	0.081
Intercept	0.782	5.199	0.882	1.685	4.791	0.728
BIS-11 at baseline	−0.042	0.086	0.632	−0.037	0.080	0.646
Leptin	0.010	0.016	0.552	0.057	0.025	0.031*
HF vs Sham	−2.518	1.645	0.136	−2.163	1.535	0.169
Intercept	2.429	5.071	0.635	2.913	5.172	0.577
BIS-11 at baseline	−0.036	0.082	0.665	−0.051	0.085	0.555
Total ghrelin	−0.051	0.075	0.498	−0.031	0.042	0.468
HF vs Sham	−3.050	1.484	0.048	−2.665	1.549	0.095
Intercept	−1.011	5.232	0.848	0.337	4.993	0.947
BIS-11 at baseline	−0.011	0.081	0.890	−0.010	0.082	0.907
Β-Endorphins	2.864	3.000	0.347	−3.945	6.379	0.540
HF vs Sham	−3.271	1.476	0.034	−3.436	1.473	0.026
Intercept	1.825	4.755	0.703	1.806	4.783	0.708
BIS-11 at baseline	−0.041	0.076	0.596	−0.038	0.077	0.627
Insulin	0.022	0.032	0.503	−0.027	0.041	0.511
HF vs Sham	−3.843	1.392	0.009	−3.724	1.432	0.013
Intercept	7.750	4.560	0.097	2.448	5.130	0.636
BIS-11 at baseline	−0.083	0.072	0.258	−0.045	0.084	0.601
Cortisol	−8.830	3.012	0.006**	6.226	4.820	0.205
HF vs Sham	−3.222	1.261	0.015	−3.980	1.489	0.011
Intercept	−0.236	5.049	0.963	2.147	4.921	0.665
BIS-11 at baseline	−0.045	0.075	0.558	−0.045	0.079	0.573
Prolactin	0.163	0.121	0.186	−0.012	0.139	0.933
HF vs Sham	−3.801	1.356	0.008	−3.470	1.424	0.020
Intercept	2.154	4.648	0.646	2.590	4.696	0.584
BIS-11 at baseline	−0.085	0.083	0.309	−0.050	0.078	0.529
FCQ	0.022	0.021	0.292	0.001	0.023	0.972
HF vs Sham	−3.685	1.361	0.010	−3.600	1.379	0.013

*HF* High Frequency group, *BMI* Body Mass Index, *BIS-11* Barratt Impulsiveness Scale-11, *FCQ-T* Food Cravings Questionnaire-Trait

*p < 0.05; ** *p* < 0.01

### Relationships between BIS-11 variation and baseline values of neuroendocrine parameters

Multivariate generalized linear regression model showed a significant inverse association between BIS-11 score change and baseline value of salivary cortisol: at increasing values of cortisol, significant lower changes in BIS-11 correspond (est −8.830, StErr 3.012, *p* = 0.006), adjusting for baseline values and treatments arms (Table 4).

In the various multivariate models, results do not change when adjusting for the sex variable.

## Discussion

Findings of the study confirmed a significant reduction of body weight and highlighted a decrease of impulsivity in individuals with obesity treated with real stimulation. A significant positive correlation between impulsivity and leptin levels reductions was found in HF. Moreover, higher cortisol levels at baseline appeared to exert a negative impact on impulsivity decrease. Together these findings allow to hypothesize that the modulation of several neuroendocrine parameters, is one of the mechanisms through which dTMS induces eating behavior control and hence, body weight loss.

The main finding of our study supports the evidence that impulsivity is closely related to the eating behaviors, playing a key role in the etiology and maintenance of obesity. Impulsivity can be conceptually divided into two forms: “stopping” impulsivity, defined as an impaired response inhibition, and “waiting” impulsivity, inability to wait or tolerate delayed rewards [26]. The PFC plays a role in modulating functions such as inhibitory control, attention, planning, risk taking, and delay discounting [27]. Neuroimaging studies demonstrated that hypoactivity of the PFC may affect inhibitory control, leading to a greater cognitive and motor impulsivity [28], namely “stopping” impulsivity. Within the PFC, the OFC appears to be mainly critical for the regulation of impulsive choices and reward-related behaviors [29]. In fact, the OFC along with the ventral striatum (specifically, the nucleus accumbens), amygdala, hippocampus, forms part of the limbic system, and is involved in an impaired reward response in impulsive individuals (“waiting” impulsivity). Alterations in these neurobiological circuits involve foremost monoaminergic signaling [e.g., dopaminergic (DA) and serotonergic (5HT) systems] [30]. Several neuroimaging studies highlighted a link between low striatal D_2/3_ receptor availability, and elevated levels of self-report and laboratory-assessed impulsivity measures, both in normal healthy volunteers and in patient population (e.g., substance-addicted individuals) [31]. This relationship appears to be mediated, in part, by diminished inhibitory autoreceptor control over stimulated striatal DA release [31]. In the context of obesity, impulsivity represents one of the possible causes leading an individual to yield to temptetion of rewarding food cues.

As well as in impulsive individuals, a lower activation in the PFC, specifically of the dorsolateral PFC (DLPFC), in response to food-related stimuli has been identified in obese compared to lean individuals [9]. Furthermore, subjects with obesity exhibit a lower sensitivity of DA-based regions, most likely due to a reduced striatal DA D_2_ receptor availability; therefore, they seek overeating to compensate for this deficiency [15]. In a recent randomized clinical trial, we demonstrated the safety and efficacy of dTMS in decreasing body wt with a long-lasting effect (up to 1 year) in individuals with obesity, suggesting as possible underlying mechanisms, the HF dTMS-induced enhancement of inhibitory capacity of PFC, and the modulation of the cortico-mesolimbic dopamine system, or “reward system” [16, 18]. In the current study, a significant decrease of body wt as well as a significant reduction in impulsivity were demonstrated after 5 weeks of dTMS treatment in HF compared to Sham group. These findings suggest a role of HF dTMS, targeted to the bilateral PFC, in modulating impulsivity, a psychological trait tighly associated with obesity, and a possible contribution of impulsivity decrease in promoting wt loss.

The evidence that anti-obesity treatments capable of modulating impulsivity are effective in promoting weight loss is sustained by studies showing, for example, the efficacy of naltrexone/bupropion, a conventional anti-obesity drug, in affecting brain’s reward system and hypothalamic pro-opiomelanocortin neurons in the Prader–Willi Syndrome, producing a synergistic effect in decreasing impulsive behavior, typical hyperphagia and body weight over time [32]. Also, lorcaserin and other 5-HT2C receptor agonists revealed effective in controlling body weight in individuals with obesity characterized by overeating due to maladaptive impulsivity and reward mechanisms [33].

No significant correlations were found between changes in body wt/BMI and the FCQ-T total score (and its subscales). The absence of correlations with other components of craving (e.g., cues, thoughts, emotions, intention, guilt) could be explained by the fact that subjects with eating disorders, according to the DSM-5 criteria (e.g., bulimia nervosa, binge-eating disorder), were not included in our clinical trial, therefore the psychopathological components of craving, usually associated with food addiction, were less significant in our sample.

The mechanisms underlying the relationship between the body wt loss and variations in psychological traits in individuals with obesity treated with dTMS, imply not only a direct effect of neurostimulation on specific brain areas, but also a possible modulation of the hormones implicated in appetite and metabolism regulation. Specifically, in this study a significant positive relationship between the variations in leptin and BIS-11 score was found in HF. Leptin is an adipose-derived peptide hormone in direct proportion to amount of body fat. It plays a significant role in food intake and energy storage regulation by relaying information between peripheral tissue and the central nervous system [34]. In normal conditions, leptin acts as an anorexigenic hormone, signaling satiety at the hypothalamic level; higher levels of leptin typically reduce appetite and food intake [35]. Therefore, the hypothesis was that leptin would provide an effective anti-obesity therapy, but this link was not confirmed by robust scientific evidence. In fact, regardless of fasting or satiety conditions, individuals with obesity exhibit elevated level of leptin due to a reduced sensitivity to leptin signaling (i.e., leptin resistance) [34], with important physiological implications.

Research over the past few years suggested that leptin and leptin resistance are associated with increased insulin resistance [36], increased systemic inflammation (TNF-α, IL6), altered modulation of pathways implicated in oncogenesis (e.g., JAK/STAT) and angiogenesis (e.g., VEGF), representing a considerable risk factor for the development of a large variety of malignancies (breast, thyroid, endometrial and gastrointestinal) [37].

In addition, leptin was shown to bind specific receptors on DA neurons in the ventral tegmental area (VTA) [37], inhibiting dopamine signaling in the nucleus accumbens [38], interacting with mesolimbic reward pathways [39], and increasing the reward value of external stimulation [40]. Elevated leptin levels are associated with craving in addictive behaviors [41–43], as well as with food cue–induced brain activations in individuals with obesity [44]. In fact, these conditions, especially drug and food craving, are typically characterized by increased impulsivity.

In our study, the finding of a direct correlation between changes in leptin levels and impulsivity is in line with a previous study highlighting in 5214 participants that some personality traits, such as impulsivity, are most consistently related to obesity and higher levels of leptin [34]. Specifically, in this study individuals with lower conscientiousness and higher impulsivity, exhibit higher circulating leptin levels, even after controlling for BMI, waist circumference or inflammatory markers [34].

In our study, a 5-week treatment with HF TMS was shown to have a significant effect on the changes in body weight and impulsivity in individuals with obesity, but not on the leptin level changes. The occurrence of a positive relationship between impulsivity variation and leptin variation (at increasing values of leptin, significant greater change in impulsivity corresponds) does not allow us to claim that reduction in impulsivity is certainly caused by the variation in leptin levels, but an influence of leptin on impulsivity could be hypothesized for an extra-hypothalamic action, in association with other factors. In summary, the evidence of this correlation leads to suggest that one of the possible mechanisms by which TMS acts is the reduction of the impulsive component associated with overeating behavior, through a modulation of leptin levels, mainly for an extra- hypothalamic TMS-induced effect on the mesolimbic reward system.

Nowadays, neither subclinical nor clinical substantial data on possible modulatory effects of neurostimulation techniques on leptin are available. Only one study demonstrated the efficacy of chronic vagus nerve stimulation in reducing leptin, together with body fat, cholesterol and triglycerides levels in rats fed a high-fat diet [45].

Also ghrelin, the principal orexigenic hormone produced by the stomach and known to increase food reward behavior, has been found to be involved in impulsive behavior. Specifically, ghrelin increases impulsivity, and changes two major components of impulsivity: motor and choice impulsivity [46]. However, in our study, no correlations between BIS-11 score and ghrelin changes have been found. The lack of this correlation may have several explanations: unlike leptin, which does not change acutely before the meals, but shows more gradual variations, ghrelin levels are characterized by considerable variability, especially related to meals. Therefore, the chronic dTMS-induced effects on ghrelin levels may be not perceivable. Furthermore, although ghrelin crosses the bloodbrain barrier, it serves as a peripheral signal to stimulate feeding, informing the arcuate nucleus of central nervous system, mainly via autonomic system (vagus nerve), which is not directly stimulated by dTMS [47]. Finally, in our study we did not measure acyl-ghrelin but total ghrelin; acetyl ghrelin, also known as circulating, represents the active form of the hormone, and is involved in promoting food intake and decreasing fat use or energy expenditure. Furthermore, in our study an inverse relationship between baseline salivary cortisol levels and change in impulsivity arose. A hyperactivation of HPA axis in obesity is well known by now and represents a prolonged adaptive response to long-term enviromental stressful events [14]. Based on preclinical studies, chronic exposure of the brain to glucocorticoids has been hypothesized to exert excitatory effects, by increasing the expression of Corticotropin Releasing Factor mRNA in the brain, which, in turn, induces recruitment of a chronic stress-response network. Moreover, glucocorticoides stimulate behaviors that are mediated by DA mesolimbic “reward” pathways, promoting, together with insulin, pleasurable and impulsive actions, such as seeking comfort foods [48]. Therefore, higher levels of cortisol, even if not at pathological levels, can account for a greater difficulty in losing body weight, due to a more impulsive psychological trait. Although our study did not reveal significant TMS-induced changes in HPA-axis hormone levels, several neurostimulation techniques have been shown effective in influencing HPA-system sensitivity, reducing cortisol levels. Modulation of the HPA axis response to stressful events, including through neurostimulation techniques, could represent a new treatment target for impulsive behaviors, disordered eating and obesity, although clinical trials focused on this outcome are needed.

Concerning the effects of dTMS on anxiety symptoms in obesity, no significant variations of STAI-*y*1 and STAI-*y*2 scores have been found in HF compared to Sham. Data on the effectiveness of TMS in anxiety are still limited and debatable because of few studies, with small samples and different study designs and protocols. A recent meta‐analysis concluded for an overall positive therapeutic effect of high‐frequency TMS to the right DLPFC for generalized anxiety disorder [49]. As previously mentioned, a possible explanation of the absence of a consistent effect of dTMS on anxiety, as well as on depression symptoms (BDI score), is the exclusion from the clinical trial of subjects with psychiatric diagnosis according to the DSM-5 criteria, and the lack of more pronounced psychopathological symptoms.

The present study has some limitations. The low number of individuals with obesity enrolled and the high number of analyzed variables could make the study results not very robust, although the rational is solid. However, future wider studies are needed to confirm the findings of this pilot study.

In conclusion, the analysis of psychological traits associated with obesity (anxiety, depression, impulsivity, different aspects of food craving) before and after a 5-week treatment with HF dTMS, highlighted a decrease of impulsivity in the individuals with obesity treated with real stimulation. This reduction together with BMI decrease in HF group suggest that impulsivity could be related to overeating and therefore, may be a risk factor for wt gain and obesity. In this study, treatment with dTMS has proven to be effective in reducing both BMI and impulsivity by enhancing inhibitory capacity of PFC and modulating several neuroendocrine parameters, in particular leptin.

## Supplementary Information

Supplementary Information

Supplementary Information

## Data Availability

Individual participant data that underlie the results reported in this paper, after deidentification (text, tables, figures, and appendices), together with the Study Protocol, will be available on demand. Data will be available for investigators whose proposed use of the data has been approved by an independent review committee (learned intermediary) identified for this purpose and for individual participant data meta-analysis.
