# Supplementary material for: Reduction of impulsivity in patients receiving deep transcranial magnetic stimulation treatment for obesity
Source: Endocrine. 2021 Jun 25;74(3):559–70. doi: 10.1007/s12020-021-02802-1 (PMC8571225; doi:10.1007/s12020-021-02802-1)
Supplement: Supplementary file 2 — Supplementary Information [file 12020_2021_2802_MOESM2_ESM.docx]

**SUPPLEMENTARY INFORMATION**

**Details and features of psychometric tests**

*The Food Cravings Questionnaire-Trait (FCQ-T) and subscales*

The food craving was assessed by the Food Cravings Questionnaire-Trait (FCQ-T), a self-report inventory. The Italian version of FCQ-T was validated by Innamorati and colleagues (2014). It is a multidimensional questionnaire consisting of 39 items selected from the literature on addiction and eating disorders; it measures nine dimensions of food craving: (a) anticipation of positive reinforcement as a result from eating (Ant+); (b) anticipation of relief from negative states and feelings as a result from eating (Ant–); (c) intentions and plans to consume food (Intent); (d) cues that might trigger food cravings (Cues); (e) thoughts or preoccupation with food (Thoughts); (f) craving as hunger (Hunger); (g) lack of control over eating (Control); (h) emotions that might be experienced before or during food cravings or eating (Emotions); and (i) guilt from cravings and for giving in to them (Guilt).

While total FCQ-T score could be used as a general measure of trait craving, individual FCQ-T factor scores could be useful in identifying and differentiating craving profiles between specific populations. FCQ-T showed a good test-retest reliability, resulting sensitive to changes in eating behavior, therefore, it has been deemed useful to detect variations of food craving in response to repetitive dTMS effect.

*Barratt Impulsiveness Scale (BIS-11)*

The Barratt Impulsiveness Scale-11 (BIS-11) questionnaire, validated into Italian language, was used to assess impulsivity. The BIS-11 is a self-report scale composed by 30 items; each item is scored 1, 2, 3, or 4, where 4 indicates the most impulsive response. The total score ranges between 30 and 120, with no established cut-off point.

State and Trait Anxiety

Inventory (STAI)

State and Trait Anxiety

Inventory (STAI),

State and Trait Anxiety

Inventory (STAI),

State and Trait

*State and Trait Anxiety Inventory (STAI)*

State and Trait Anxiety Inventory (STAI) test was used to assess anxiety. The test includes two axes (*y*1 for state anxiety and *y*2 for trait anxiety), both consisting of 20 multiple-choice items; each item has a score from one to four, and he total point score of *y*1 and *y*2 axes can range from 20 to 80 (Spielberg et al, 1983). A threshold of 40 was used to distinguish between high- and low-anxiety.

State anxiety is defined as an emotive state of apprehension and tension, perceived on a conscious level, variable over time and influenced by temporary stressful situations; anxiety as a trait refers to individuals with continuous disposition towards anxiety.

*Beck Depression Inventory*

The Beck Depression Inventory (BDI) is the most frequently used measure of depression in the literature, especially as measure of coping. The original version of BDI consists of 21 items which respondents rate on a scale from 0 to 3. In this study, the short-form 13-item version of the BDI was used.

**Details of laboratory measurement procedures**

Insulin, TSH, prolactin, salivary cortisol were determined with the electrochemiluminescence immunoassay (ECLIA). Total ghrelin and β-endorphins levels were measured using commercially available enzyme immunoassay (EIA) kits (Phoenix Pharmaceuticals, Burlingame, CA, USA); enzyme-linked immunosorbent assay (ELISA) kits were used to assess epinephrine, norepinephrine (Elabscience Biotechnology Co. Ltd, Wuhan, China) and leptin (Diagnostic Biochem Canada Inc, London, Ontario, Canada). Glucagon was assessed by the radioimmunoassay (RIA) and glucose by the UV enzymatic method with hexokinase.

**Details of dropped-out patients**

Four patients decided to withdraw from the study for personal reasons other than side effects (2 in HF, 2 in Sham), one patient (in HF) discontinued the treatment for a possible treatment side effect (vaso-vagal reaction), another patient reported an asymptomatic incidental meningioma (in HF) diagnosed while performing a head MRI. Dropout patients were excluded from the statistical analysis.
